# Psychotherapist Trainees’ Quality of Life: Patterns and Correlates

**DOI:** 10.3389/fpsyg.2022.864691

**Published:** 2022-03-24

**Authors:** Erkki Heinonen, David E. Orlinsky, Ulrike Willutzki, Michael Helge Rønnestad, Thomas Schröder, Irene Messina, Henriette Löffler-Stastka, Armin Hartmann

**Affiliations:** ^1^Department of Psychology, University of Oslo, Oslo, Norway; ^2^Finnish Institute for Health and Welfare, Helsinki, Finland; ^3^Department of Comparative Human Development, University of Chicago, Chicago, IL, United States; ^4^Department of Psychology and Psychotherapy, University of Witten/Herdecke, Witten, Germany; ^5^Division of Psychiatry and Applied Psychology, University of Nottingham, Nottingham, United Kingdom; ^6^Universitas Mercatorum, Rome, Italy; ^7^Department of Psychoanalysis and Psychotherapy, Medical University of Vienna, Vienna, Austria; ^8^Department of Psychosomatic Medicine and Psychotherapy, Faculty of Medicine, Medical Center – University of Freiburg, Freiburg im Breisgau, Germany

**Keywords:** psychotherapist training, psychotherapists, life quality, life satisfaction, life stress, relationships

## Abstract

While psychotherapists are trained to improve their clients’ quality of life, little work has examined the quality of life experienced by psychotherapist trainees themselves. Yet their life satisfactions and stresses would plausibly affect both their ability to learn new skills and conduct psychotherapy. Therefore, in the Society for Psychotherapy Research Interest Section on Psychotherapist Development and Training study, we investigated the patterns of self-reported life quality and their correlates in a multinational sample of 1,214 psychotherapist trainees. A comprehensive questionnaire was used at the outset of trainings to assess trainees’ professional background, current life situation, personal characteristics, family background, and social and national origin. The findings indicated 54.3% of trainees’ lives could be characterized as fortunate or happy (i.e., experiencing great life satisfaction and not much stress), whereas 14.3% could be characterized as clearly distressed or troubled (i.e., experiencing great life stress and not much satisfaction). The strongest correlates of high life stress, a contributor to poor life quality, were economic insecurity, self-protectiveness, and attachment-related anxiety in relationships, and economic or psychological hardship in childhood. In turn, greater wellbeing was most strongly associated with a warm and open interpersonal style, being married, having sufficient economic means, and material and emotional security in childhood. While the results indicate the majority of therapists experience a relatively good quality of life, the findings also suggest potential targets for increasing trainees’ life quality when it may be deficient, such as those on a societal level (e.g., availability of low-cost student loans), training program level (e.g., promoting supportive supervision, positive between-trainee relationships and group collaboration), and individual level (e.g., personal therapy and learning self-care), in order to promote effective learning and therapy practice.

## Introduction

The work that psychotherapists do with clients generally aims to improve their clients` quality of life, and researchers have invested decades of work to understand the circumstances in which, and processes though which, such improvement occurs (e.g., Garfield and Bergin, 1971; Barkham et al., 2021). Until recently, much less interest has focused on the quality of life experienced by psychotherapists themselves, although it may be one of the factors that contribute to their influence on clients (e.g., Guy et al., 1989; Schröder et al., 2009; Nissen-Lie et al., 2013; Heinonen and Nissen-Lie, 2019; Brugnera et al., 2020). Even less studied has been the life quality of psychotherapist trainees, despite the fact that many are young adults who in general have been found vulnerable to mental health issues (Kessler et al., 2005). Other studies suggest that stress may be a particular problem for students in the caring professions, such as medicine (Dahlin et al., 2005) and nursing (Nerdrum et al., 2009). The present study examines these issues by exploring the patterns and correlates of life quality among individuals who are currently in training to become psychotherapists.

Generally, life satisfaction (or subjective wellbeing) has been shown to correlate positively with work performance and negatively with absenteeism (Tenney et al., 2016). Among psychotherapists, practitioners with greater life satisfaction experience themselves as more effective (Beutler et al., 2004; Orlinsky, 2022). By contrast, therapist burnout, which is likely associated with a lower overall quality of life, has been linked to worse treatment outcomes for patients (Beutler et al., 2004; Steel et al., 2015; Delgadillo et al., 2018). As for psychotherapy trainees, the quality of their personal lives would likely affect their ability both to learn new skills and to perform well in clinical settings (e.g., Beaumont et al., 2016; Bücker et al., 2018; Messina et al., 2019; Chattu et al., 2020).

Previous studies have solidly established one source for therapists’ decreased life satisfaction, i.e., work-related stresses contributing to professional burnout. A recent meta-analysis of relatively experienced therapists identified such expectable risk factors for burnout as longer working hours, role overload and role conflict, high caseload, and negative clientele (Lee et al., 2020). Another review identified major burnout risk factors plausibly affecting especially early career therapists, i.e., younger age, less work experience, and being overinvolved in client problems (Simionato and Simpson, 2018). Of note, a few studies in the review also identified dispositional risk factors for burnout, such as neuroticism, perfectionism, low agreeableness, and low extraversion. A third critical review underlined the particular needs of newly qualified counselors and psychotherapists, such as adequate mentoring and supervision, to counter work-related stresses like professional self-doubt and vicarious traumatization which may predispose to burnout (Davies et al., 2021).

While there is substantial knowledge of work-related correlates of professional distress (many plausibly affecting especially younger or less experienced therapists), little research to date has focused on future therapists’ wellbeing overall—i.e., comprising both the satisfactions and the stresses they experience in their lives—as well as their circumstances. Prior literature suggests that life satisfaction varies considerably and that important correlates comprise relatively stable individual characteristics, such as personality and its temperamental and genetic determinants (Schimmack et al., 2004; Steel et al., 2008), early life experiences (Frijters et al., 2014); current life circumstances, such as financial situation (Lucas and Schimmack, 2009) and social support (Myers, 2000); sociodemographic variables, such as age (Diener et al., 2018) or minority vs. majority status (Veenhoven and Hagerty, 2006); and some broader cultural and environmental factors, like those related to nationality (e.g., Oishi et al., 1999; Delle Fave et al., 2016).

Conceptually, overall quality of life reflects a balance between the levels of satisfactions and stresses in a person’s current life. Satisfaction and stress are far from polar opposites, although extremes of both are unlikely to coexist. Low or even relatively high levels of satisfaction can co-occur with low or even relatively high levels of stress, and the levels of each relative to the other will define *qualitatively different patterns* of life quality. Such patterns will constitute the criterion variable in our study, and various factors in the lives of a large multinational sample of trainees will be explored as potential predictors of those patterns (and/or the ratings of life satisfaction and stress levels that constitute the patterns). Knowledge of the impact of such characteristics in therapist trainees could serve: (1) to illuminate the general status of future therapists’ wellbeing, and both the protective and risk factors associated with it; (2) to help identify trainees for whom a supportive work environment might be especially helpful, or a challenging environment especially burdening; and (3) to identify protective or risk factors that could be modified to support psychotherapy trainees’ wellbeing, learning, and effective practice.

### Research Question

Based on the prior literature reviewed above, the research question of our study was: How is the quality of life of psychotherapist trainees associated with their (1) current life situation; (2) personal characteristics; (3) family background; and (4) national and social origins?

## Materials and Methods

### Design

This study reports on one aspect of a broader collaborative international longitudinal study of psychotherapy training that has been conducted by members of the Society for Psychotherapy Research Interest Section on Psychotherapist Development and Training (SPRISTAD; Orlinsky et al., 2019). From 2016 to the present, the SPRISTAD collaborative has collected information about psychotherapy training programs and trainees from different professions and theoretical orientations in many countries, in a conceptually organized exploratory and inductive study of the features of training programs, and the corresponding formative experiences, practices, and development of trainees. The study instruments focus mainly on trainees’ professional characteristics, activities, and development but also solicit information on aspects of their personal lives. Overall, the principal goals of the SPRISTAD study are: (1) identifying common and divergent features of psychotherapy training programs; (2) tracking progressive changes over time in trainees as therapists; (3) identifying factors that tend to facilitate or impede trainee development; (4) using quantitative and qualitative data gathered from a large number of psychotherapy trainees of varied types in a wide range of training programs.

Training centers and trainees collaborating in the study have been recruited through professional publications, workshops and conferences, professional societies and individual collegial networks. Training centers that participate in the SPRISTAD study have a local research coordinator who is a SPRISTAD member and must offer training programs of at least 12 months’ duration. Assessments with various instruments are made at the start of the trainee’s program, after 6 months, and after 12 months. Therapist trainees in the present study were informed about the study by their local research coordinators at the beginning of their training, and those who gave informed consent were provided online with the SPRISTAD questionnaires at the start of their training from the data collection center at Witten/Herdecke University in Germany; or, in the case of Finland, through the Finnish research coordinator; or, in the case of a subset of Italian participants, *via* a paper-and-pencil measure through the local research coordinator. The present study is based on data collected to date from countries that had at least 10 trainees who responded to the first SPRISTAD research measure.

### Measures

#### Trainee Background Information Form

Data for the present study were collected with the *Trainee Background Information Form* (TBIF), which is a survey of trainees’ demographic, professional, and familial backgrounds, and some personal psychological characteristics, that trainees complete when starting on their training program. Most items are structured-response scales or checklists, but space is provided for textual response to open-ended questions. The TBIF draws on items from the *Development of Psychotherapists Common Core Questionnaire* (DPCCQ), which has a current worldwide data base of about 12,000 psychotherapists (Orlinsky and Rønnestad, 2005; Orlinsky, 2022).

The domains of the TBIF relevant to present study include trainees’ *demographic* information (age, gender, country of residence, immigration status, and minority vs. majority status); their *training level* (years of prior therapy practice, if any); their *current life situation* (marital status, parental status, and financial status); and their *family background* (family size, trainee birth order, family material wellbeing, and family emotional/psychological functioning). For the latter two, trainees are asked to report on the material and economic circumstances of their family-of-origin, on a 5-point scale from “Very comfortable” to “Marginal”; and to rate how well their childhood family functioned psychologically and emotionally, rated on a 6-point scale (anchored as 0 = “not at all,” 1 = “little,” 2 = “some,” 3 = “moderately,” 4 = “greatly,” 5 = “very greatly”).

##### Adult Attachment

Adult attachment was operationalized using an adaptation of Wei et al. (2007) short version of the *Experiences in Close Relationships* questionnaire (ECR-S). The questionnaire yields scores on two dimensions of insecure (*vs* secure) insecure (*vs* secure) attachment: Avoidant Attachment (six items, e.g., “I prefer not to show a partner how I feel deep down”) and Anxious Attachment (six items, e.g., “I worry about being abandoned”). Items are rated on a Likert-type scale ranging from 1 (strongly disagree) to 7 (strongly agree). These subscales have shown good test–retest reliability (Wei et al., 2007), between 0.80 and 0.89 in different sub studies, and sufficient to good internal consistency: *α* = 0.78 (Anxiety); *α* = 0.84 (Avoidance). Comparable internal consistency of dimensions was found for the current trainee sample: *α* = 0.74 (Anxiety); *α* = 0.80 (Avoidance).

##### Personal Identity

Trainees’ personal identity, defined as their self-experience in close personal relationships, was assessed in the TBIF using 35 7-point self-descriptive adjectival items (anchored at 0 = “not at all,” 2 = “some,” 4 = “much,” 6 = “very much”), presented following the question: “How would you describe yourself as you really are in your close personal relationships?” Interpersonal aspects of self were assessed with items based on Leary’s (1957) circumplex model of interpersonal behavior. Temperament aspects of self were assessed with items reflecting amplitude vs. restraint in individual’s cognitive-instrumental and emotional-expressive functioning. Exploratory factor analysis of these items (principal components extraction, Varimax rotation) yielded four dimensions that essentially replicated prior similar factor analyses on a sample of over 10,000 graduated and practicing therapists (Orlinsky et al., 2019; Orlinsky, 2022). Reliable multiple-item scales were constructed for the following four dimensions: (1) *Genial/Caring*, consisting of seven adjective items (warm, friendly, tolerant, receptive, nurturant, optimistic, accepting; *α* = 0.76) viewed as *self-bestowal*; (2) F*orceful/Exacting*, consisting of five adjective items (directive, demanding, authoritative, challenging, critical; *α* = 0.76), viewed as *self-assertion*; (3) *Reclusive/Remote*, consisting of four adjectives (reserved, guarded, private, skeptical; *α* = 0.72), viewed as *self-protection*; and *Practical/Determined*, also consisting of four adjectives (organized, pragmatic, determined, energetic, *α* =0.66), viewed as *self-efficacy*. Alpha coefficients for these multi-item scales were considered adequate in light of the number of items used in each and their highly significant correlations with other therapist characteristics.

##### Life Satisfaction and Stress

The criterion variable for this study was derived from two questions in the TBIF: “How satisfying is your life at present?” and “How stressful is your life at present?” Response alternatives for each used a 6-point scale (rated 0 = “not at all,” 1 = “little,” 2 = “some,” 3 = “moderately,” 4 = “greatly,” 5 = “very greatly”).

### Sample

The sample for this study comprises 1,214 psychotherapist trainees, whose demographic and professional characteristics are summarized in Table 1. Approximately three quarters of the total come from five European countries (Finland, Austria, Italy, Germany, and United Kingdom). About 85% are female. The average age of the group was 36 (*M* = 35.8, SD = 9.3) but the age range was substantial (19–71, with most between 27 and 45 years of age). About one in 10 identified with either a minority or immigrant status. Over four out of five trainees viewed themselves as training professionally in the field of psychotherapy, two-thirds at the initial level of professional training (0 to <2 years of practice) and one-third at an advanced level of training. All major therapy orientations were represented in the trainings.

**Table 1:** Trainees’ demographic and professional training characteristics (*N* = 1,214).^1^

	*N*	%		*N*	%	
**Nation**			**Gender**			
Argentina	44	3.6%	Female	1,055	84.5%	
Austria	270	22.2%	Male	194	15.5%	
Canada	11	0.9%	**Age** (years)			
Chile	60	4.9%	*M* = 35.8	Med = 33.3		
Finland	242	19.9%	SD = 9.3	Range 19.6–70.4		
Germany	141	11.6%	**Age group**	*N*	*%*	
Italy	231	19.0%	19–29	424	35.3%	
Lithuania	31	2.6%	30–39	417	34.7%	
Romania	12	1.0%	40–71	361	30.0%	
Switzerland	35	2.9%	**Social marginality**			
United Kingdom	123	10.1%	Minority	88	7.5%	
United States of America	14	1.2%	Immigrant	123	10.2%	
**Perceived professional field** ^2^		*N*	%	**Perceived program orientation** ^3^	*N*	%
Psychotherapist		796	83.0%	Analytic/Psychodynamic	444	36.9%
Psychologist		58	6.1%	Behavioral	300	25.0%
Counselor		35	3.7%	Cognitive	379	31.5%
Psychoanalyst		36	3.8%	Cognitive-Behavioral^5^	310	25.8%
Marital therapist/counselor		33	3.4%	Humanistic	390	32.4%
				Interpersonal	410	34.1%
**Prior therapy practice** ^4^				Systemic	263	21.9%
None		609	60.5%	Integrative	436	36.3%
>0 to 1 year		33	3.2%	No salient orientation	39	3.2%
>1 to 2 years		37	3.7%	**Professional training level**		
>2 to 5 years		128	12.7%	Initial Training (0–2 years)	679	67.5%
>5 years		200	19.8%	Advanced Training (>2 years)	328	32.5%

1 Ns vary slightly due to occasional missing data.

2 Multiple ratings allowed; *N* = 958.

3 “Salient” program orientation rated ≥ 8 on 0–10 scale of influence; multiple ratings allowed; Med = 2.

4 *N* = 1,006.

5 Computed as: Cog + Behav/2.

Trainees’ current life status and family backgrounds are summarized in Table 2. About three fifths were in a committed adult relationship, with just over one-third married, another one-fourth living with their partner, and with the remainder as single, either unattached (17.7%) or in a relationship (17.9%). In terms of family background, 83.9% had siblings (mostly just one or two). Approximately a quarter of the trainees reported experiencing serious financial difficulty, but a majority had either no or only slight economic difficulties. A majority grew up in economically comfortable families, but over 1 in 7 experienced economic hardships in their childhood families.

**Table 2:** Trainees’ current life status and family background.

Marital status	*N*	%^1^	Current economic/financial situation	*N*	%^2^
Single (unattached)	205	17.7%						
Single (in a relationship)	207	17.9%	Not at all difficult	288	24.1*%*
Living with partner	321	27.7%	Slightly difficult	361	30.2*%*
Married/remarried	391	33.8%	Somewhat difficult	222	18.6*%*
Divorced/separated	33	2.9%	Moderately difficult	225	18.8*%*
Parental status			Very difficult	81	6.8*%*
Have children:	439	36.4%	Extremely difficult	19	1.6*%*
Family of origin	*N*	*%* ^3^	Family economic level	*N*	*%* ^4^
Only child	159	16.1*%*	Very comfortable: we had all that we wanted	173	14.4%
2 child family	403	40.7*%*			
3 child family	241	24.4*%*	Comfortable: we had all we needed and some extra	508	42.3%
4+ child family	186	18.8*%*			
Oldest of 2+ children	330	33.4*%*^5^	Sufficient: had all we needed	342	28.5%
Middle of 3+ children	210	21.28*%*	Insecure: occasional stress	148	12.3%
Youngest of 2+ children	292	29.5*%*	Marginal: real hardship	29	2.4%

1 Based on *N* = 1,178.

2 Based on *N* = 1,214.

3 Based on *N* = 989.

4 Based on *N* = 1,200.

5 Based on *N* = 989 (*N* = 832, excluding only children).

### Ethics

Upon participating in the study, the local training centers addressed their respective research ethics committees and received ethical approval. All trainees were given written information about the study and signed an informed consent document.

### Statistical Analysis

Chi-square tests were used to determine the association between categorical variables. Non-parametric Pearson correlations (*ρ*) were calculated to determine the association between continuous measures for Personal Self and Adult Attachment and the Life Quality component variables (Life Satisfaction and Life Stress). This first exploratory investigation has assessed relationships with multiple independent variables, without an alpha-level adjustment, since detecting potential factors related to trainee life quality is the principal task, rather than hypothesis testing. Analyses were conducted using SPSS for Mac version 28.0.0.

## Results

### Trainees’ Life Satisfaction and Stress and Their Patterns

With regard to the criterion variable, Table 3 shows that almost two-thirds of the trainees reported experiencing “great” or “very great” current life satisfaction, whereas just under one-fourth reported experiencing “great” or “very great” life stress. However, 50.3% of the trainees reported “some” or “moderate” stress in their lives, indicating that meaningful levels of stress were present for about three-fourths of them.

**Table 3:** Trainees’ current life satisfaction, life stress, and life quality patterns.

How satisfying is your life at present?	*N*	*%*	How stressful is your life at present?	*N*	*%*
0—Not at all	3	0.3*%*	0—Not at all	43	3.6*%*
1—Little	29	2.4*%*	1—Little	275	23.1*%*
2—Some	74	6.2*%*	2—Some	274	23.0*%*
3—Moderately	325	27.1*%*	3—Moderately	325	27.3*%*
4—Greatly	604	50.3*%*	4—Greatly	235	19.7*%*
5—Very greatly	165	13.8*%*	5—Very greatly	39	3.3*%*
Total	1,200	100.0*%*	Total	1,191	100.0*%*
	**Life quality patterns** (*N* = 1,191)				
**Current life satisfaction**	**Current life stress**				
	Less than great (0–3)		Great/Very great (4–5)		
Great/Very great (4–5)	**Fortunate/Happy**54.3% (*n* = 659)		**Intense/Impassioned**8.7% (*n* = 104)		
Less than great (0–3)	**Low-key/Subdued**21.7% (*n* = 258)		**Distressed/Troubled**14.3% (*n* = 170)		

For most of the subsequent analyses, high and low levels of life satisfaction and stress were combined to define four distinct life quality patterns (Table 3). A majority (54.3%) of the trainees evidently had *Fortunate/Happy* lives [great or very great satisfaction with no more than moderate stress. By contrast, just over one-fifth (21.7%) of the trainees’ seemed to be leading rather *Low-Key/Subdued* lives (no great satisfaction, and no great stress]. The remaining one-fourth of the sample was divided between two smaller patterns: Currently the lives of about one in 11 (8.7%) of the trainees could be described as *Intense/Impassioned* (having both great satisfaction and great stress), while even more (14.3%) of the trainees appeared to be living a *Distressed/Troubled* life (experiencing great stress and no great satisfaction). The next step was to determine which if any personal and life characteristics are associated with the trainees’ varied quality of life.

### Current Life Situation and Current Life Quality

#### Gender and Age

Table 4 shows there were no significant differences in life quality patterns between women and men, but that Older trainees (ages 40–70) tended to experience a *Fortunate/Happy* life quality significantly more often than Mature trainees (ages 30–39) and especially more than Younger trainees (ages 19–29): 62.0% vs. 55.4% and 49.4%, respectively.

**Table 4:** Trainees’ current life quality by gender and age.^1^

Life quality pattern		Gender^2^			Age group^3^,^4^			
		Female	Male	Total	Younger (19–29)	Mature (30–39)	Older (40–70)	Total
Fortunate/Happy	*n*	555	103	658	206	227	219	652
	%	46.7%	8.7%	55.3%	(*49.4%*)	55.4%	(**62.0%**)	55.3%
Low-key/Subdued	*n*	215	42	257	105	76	74	255
	%	18.1%	3.5%	21.6%	25.2%	18.5%	21.0%	21.6%
Intense/Impassioned	*n*	88	15	103	42	38	24	104
	%	7.4%	1.3%	8.7%	10.1%	9.3%	6.8%	8.8%
Distressed/Troubled	*n*	144	26	170	64	69	36	169
	%	12.1%	2.2%	14.3%	15.3%	16.8%	10.2%	14.3%
Total	*N*	1,002	186	1,188	417	410	353	1,180
	%	84.3%	15.6%	100.0%	35.3%	34.5%	29.9%	100.0%

1 % in bold type indicates cell significantly high at *p* = 0.01 (if in parentheses, at *p* = 0.05); % in underlined italics indicates cell significantly low at *p* = 0.01 (if in parentheses, at *p* = 0.05).

2 *χ*^2^ = 0.20, df = 3, *p* = ns.

3 *χ*^2^ = 18.6, df = 6, *p* = 0.005 (“Older” more, “Younger” less Fortunate/Happy, *p* = 0.05).

4 Correlations: age × life satisfaction, *ρ* = 0.09 (*p* = 0.002); age × life stress, *ρ* = −0.09 (*p* = 0.002).

#### Marital and Parental Status

Table 5 shows that currently Married trainees experienced a *Fortunate/Happy* quality of life most often, and significantly more often than Single Unattached trainees (66.3% vs. 40.6%), while Single Unattached trainees were most likely of all groups to have a *Low-key/Subdued* life quality, and Married trainees were least likely to (36.1% vs. 14.1%). No differences were found between parents vs. non-parents within marital status categories.

**Table 5:** Trainees’ current life quality by marital/relationship status.^1^,^2^

Life quality pattern		Single unattached	Single in a relationship	Living w. partner	Married/Remarried	Divorced/Separated	Total
Fortunate/Happy	*n*	82	100	190	258	16	646
	%	*40.6%*	48.5%	59.6%	**66.3%**	48.5%	56.2%
Low-key/Subdued	*n*	73	57	54	55	10	249
	%	**36.1%**	27.7%	16.9%	*14.1%*	30.3%	21.7%
Intense/Impassioned	*n*	16	14	29	36	2	97
	%	7.9%	6.8%	9.1%	9.3%	6.1%	8.4%
Distressed/Troubled	*n*	31	35	46	40	5	157
	%	15.3%	17.0%	14.4%	10.3%	15.2%	13.7%
Total	*N*	202	206	319	389	33	1,149
	%	17.6%	17.9%	27.8%	33.9%	2.9%	100.0%

1 *χ*^2^ = 63.5, df = 12, *p* < 0.001. % in bold type indicates cell significantly high at *p* = 0.01 (if in parentheses, at *p* = 0.05); % in underlined italics indicates cell significantly low at p = 0.01 (if in parentheses, at *p* = 0.05).

2 Differences between parent vs. non-parent within marital status categories, all are *p* = ns.

#### Current Economic/Financial Circumstances

Table 6 shows that trainees’ current economic and financial circumstances are significantly associated with their current life quality. Nearly three-fourths (73.8%) of those with no economic difficulties report a *Fortunate/Happy* quality of life, as compared with only 37.5% of those with moderately difficult or very difficult circumstances. At worst, more than one-fourth (27.5%) of the latter had a *Distressed/Troubled* life quality (about 7 times more often than those with no financial difficulties), and at best another one-fourth (23.8%) had a *Low-key/Subdued* life quality.

**Table 6:** Trainees’ current life quality by economic/financial circumstances.^1^

Life quality pattern		Current economic/Financial circumstances			Total
		Not at all difficult	Slightly/Somewhat difficult	Moderately/Very difficult	
Fortunate/Happy	*n*	211	325	120	656
	%	**73.8%**	56.3%	*37.5%*	55.5%
Low-key/Subdued	*n*	41	141	76	258
	%	*14.3%*	24.4%	23.8%	21.8%
Intense/Impassioned	*n*	22	44	36	102
	%	7.7%	7.6%	11.3%	8.6%
Distressed/Troubled	*n*	12	67	88	167
	%	*4.2%*	11.6%	**27.5%**	14.1%
Total	*N*	286	577	320	1183
	%	24.2%	48.8%	27.0%	100.0%

1 *χ*^2^ = 112.3, df = 6, *p* = <0.001. % in bold type indicates cell significantly high at *p* = 0.01 (if in parentheses, at *p* = 0.05); % in underlined italics indicates cell significantly low at *p* = 0.01 (if in parentheses, at *p* = 0.05).

### Personal Characteristics and Current Life Quality

Personal SelfThe upper tier of Table 7 shows a number of significant correlations between trainees’ Personal Self dimensions and the components measures of Life Quality. (1) There was a moderate positive correlation (*ρ* = 0.26) of *Genial/Caring* (self-bestowal) with Current Life Satisfaction, and a small but significant inverse correlation (*ρ* = −0.12) with Current Life Stress. *Practical/Determined* (self-efficacy) also had a small but significant positive correlation (*ρ* = 0.13) with Current Life Satisfaction. (2) By contrast, *Reclusive/Remote* (self-protection) was modestly but significantly correlated (*ρ* = 0.19) with Current Life Stress, and negatively correlated (*ρ* = −0.16) with Current Life Satisfaction. *Forceful/Exacting* (self-assertion) also had a small but significant positive correlation (*ρ* = 0.11) with Current Life Stress.

**Table 7:** Trainees’ current life quality by personal self and attachment style.

Self in close personal relationships		How satisfying is your life?^1^	How stressful is your life?^2^
Genial/Caring (*α* = 0.74)	*ρ*	0.26^**^	−0.12^**^
Forceful/Exacting (*α* = 0.76)	*ρ*	−0.04	0.11^**^
Reclusive/Remote (*α* = 0.72)	*ρ*	−0.16^**^	0.19^**^
Practical/Determined (*α* = 0.66)	*ρ*	0.13^**^	0.03
Adult attachment style		How satisfying is your life?^3^	How stressful is your life?^4^
Anxious Attachment (*α* = 0.74)	*ρ*	−0.26^**^	0.25^**^
Avoidant Attachment (*α* = 0.80)	*ρ*	−0.18^**^	0.13^**^

** *p* < 0.001.

1 *N* = 1,190.

2 *N* = 1,181.

3 *N* = 1,189.

4 *N* = 1,180.

#### Adult Attachment

The lower tier of Table 7 shows that both Anxious Attachment and Avoidant Attachment were significantly correlated with the component Life Quality dimensions, positively with Current Life Stress (*ρ* = 0.25 and *ρ* = 0.13, respectively), and negatively with Current Life Satisfactions (*ρ* = −0.26 and *ρ* = −0.18, respectively).

### Family Backgrounds and Current Life Quality

#### Childhood Family Size

No significant associations were found between trainees’ current Life Quality and the size of their family of origin (i.e., number of sibs) or their birth order.

#### Family Backgrounds

Table 8 shows some significant associations between the trainees’ current Life Quality and the material/economic level of their childhood families. Nearly three-fourths (72.5%) of the trainees who grew up in materially very comfortable circumstances were experiencing a *Fortunate/Happy* quality of life as adults, and very few (6.4%) had a *Distressed/Troubled* life. By contrast, nearly four times as many (23.4%) of the trainees who grew up in materially insecure/marginal circumstances had a *Distressed/Troubled* quality of life as adults, and less than half (43.3%) were enjoying a *Fortunate/Happy* quality of life.

**Table 8:** Trainees’ current life quality by family economic background.^1^,^2^

Life quality pattern		Family economic background				Total
		Very comfortable: had all we wanted	Comfortable: had all we needed + some extra	Sufficient: had all we needed but just that	Insecure/Marginal: serious want and worries	
Fortunate/Happy	*n*	124	291	167	74	656
	%	**72.5%**	57.7%	49.6%	*43.3%*	55.5%
Low-key/Subdued	*n*	25	111	78	43	257
	%	14.6%	22.0%	23.1%	25.1%	21.7%
Intense/Impassioned	*n*	11	45	32	14	102
	%	6.4%	8.9%	9.5%	8.2%	8.6%
Distressed/Troubled	*n*	11	57	60	40	168
	%	*(6.4%)*	11.3%	17.8%	**23.4%**	14.2%
Total	*N*	171	504	337	171	1183
	%	14.5%	42.6%	28.5%	14.5%	100.0%

1 *χ*^2^ = 46.2, df = 9, *p* < 0.001. % in bold type indicates cell significantly high at *p* = 0.01 (if in parentheses, at *p* = 0.05); % in underlined italics indicates cell significantly low at *p* = 0.01 (if in parentheses, at *p* = 0.05).

2 Correlation of family economic background X current life satisfaction, *ρ* = 0.18 (*p* < 0.001); family economic background X current life stress, *ρ* = −0.14 (*p* < 0.001).

#### Family Emotional/Psychological Functioning

The TBIF explored trainee’s experiences of their childhood family’s emotional atmosphere by asking: “Did the family you grew up in function well emotionally and psychologically?” The response alternatives (0 = Not at all, 1 = Little, 2 = Some, 3 = Moderately, 4 = Greatly, 5 = Very greatly) were condensed for this analysis into three categories: Poor (0–2) for 30.7% of trainees, Moderate (3) for 31.1% of trainees, and Good (4–5) for 38.2% of trainees. In effect, a clear majority (61.8%) of trainees rated their childhood family’s emotional atmosphere as poor or moderate at best.

Table 9 shows that trainees who grew up in families with good emotional/psychological functioning were significantly more likely (63.4%) to have a *Fortunate/Happy* quality of life as adults, especially in contrast to those from psychologically poorly functioning families (46.2%). On the other hand, one-fifth (20.9%) of the trainees who grew up in families with poor emotional/psychological functioning were experiencing a *Distressed/Troubled* life quality, which was more than twice the proportion (9.3%) of those from well-functioning families.

**Table 9:** Trainees’ current life quality by family of origin emotional function.^1^,^2^

Life quality pattern		Family emotional and psychological functioning			Total
		Poor (0–2)	Moderate (3)	Good (4–5)	
Fortunate/Happy	*n*	168	200	288	656
	%	*46.2%*	54.2%	**63.4%**	55.3%
Low-key/Subdued	*n*	95	87	76	258
	%	26.1%	23.6%	*16.7%*	21.7%
Intense/Impassioned	*n*	25	31	48	104
	%	6.9%	8.4%	10.6%	8.8%
Distressed/Troubled	*n*	76	51	42	169
	%	**20.9%**	13.8%	*9.3%*	14.2%
Total	*N*	364	369	454	1187
	%	30.7%	31.1%	38.2%	100.0%

1 *χ*^2^ = 42.5, df = 6, *p* < 0.001. % in bold type indicates cell significantly high at *p* = 0.01 (if in parentheses, at *p* = 0.05); % in underlined italics indicates cell significantly low at *p* = 0.01 (if in parentheses, at *p* = 0.05).

2 Correlation of family emotional functioning X current life satisfaction, *ρ* = 0.22 (*p* < 0.001); family emotional functioning X current life stress, *ρ* = −0.14 (*p* < 0.001).

### Social and National Aspects of Current Life Quality

#### Social Marginality

Table 10 presents two measures of social marginality and their relationship to trainees’ Current Quality of Life. Being an immigrant vs. native-born had no relationship to trainees’ life quality. However, perceiving oneself as having a minority vs. mainstream status did. Trainees who had a mainstream social identity were significantly more likely than those with a minority identity to be experiencing a *Fortunate/Happy* life quality (56.2% vs. 38.1%). By contrast, trainees with a minority identity were significantly more likely than mainstream trainees to have an *Intense/Impassioned* quality of life (20.2% vs. 8.0%).

**Table 10:** Trainees’ current life quality by social marginality.

Life quality pattern		Minority status^1^			Immigrant status^2^		
		Minority	Mainstream	Total	Immigrant	Native	Total
Fortunate/Happy	*n*	32	604	636	68	590	658
	%	*38.1%*	**56.2%**	54.9%	57.1%	55.2%	55.4%
Low-key/Subdued	*n*	21	233	254	26	231	257
	%	25.0%	21.7%	21.9%	21.8%	21.6%	21.7%
Intense/Impassioned	*n*	17	86	103	11	93	104
	%	**20.2%**	*8.0%*	8.9%	9.2%	8.7%	8.8%
Distressed/Troubled	*n*	14	152	166	14	154	168
	%	16.7%	14.1%	14.3%	11.8%	14.4%	14.2%
Total	*N*	84	1,075	1,159	119	1,068	1,187
	%	7.2%	92.8%	100.0%	10.0%	90.0%	100.0%

1 *χ*^2^ = 18.5, df = 3, *p* < 0.001. % in bold type indicates cell significantly high at *p* = 0.01 (if in parentheses, at *p* = 0.05); % in underlined italics indicates cell significantly low at *p* = 0.01 (if in parentheses, at *p* = 0.05).

2 *χ*^2^ = 0.64, df = 3, *p* = ns.

#### National Origins

As research on life quality generally reveals significant differences between countries, we also compared trainees from the six countries with the largest representations in our sample. Table 11 shows a *Fortunate/Happy* quality of life was enjoyed by significantly large majorities of the trainees from Finland (73.1%) and Austria (65.9%), and by a significantly very much smaller proportion of trainees from Italy (27.2%). The Italian trainees were significantly more likely to be experiencing a *Distressed/Troubled* life at worst (29.5%), or a *Low-key/Subdued* life quality at best (31.7%). There was a significant tendency for one-fifth (20.0%) of the smaller group of trainees from Chile to experience an *Intense/Impassioned* life, even though the majority (56.7%) of those from Chile had a *Fortunate/Happy* quality of life.

**Table 11:** Trainees’ current life quality by nation.^1^

Life quality pattern		Austria	Chile	Finland	Germany	Italy	United Kingdom	Total
Fortunate/Happy	*n*	178	34	171	77	61	61	582
	%	**65.9%**	56.7%	**73.1%**	54.6%	*27.2%*	49.6%	55.3%
Low-key/Subdued	*n*	53	6	38	36	71	32	236
	%	19.6%	10.0%	16.2%	25.5%	**31.7%**	26.0%	22.4%
Intense/Impassioned	*n*	18	12	10	10	26	12	88
	%	6.7%	**20.0%**	4.3%	7.1%	11.6%	9.8%	8.4%
Distressed/Troubled	*n*	21	8	15	18	66	18	146
	%	*(7.8%)*	13.3%	*6.4%*	12.8%	**29.5%**	14.6%	13.9%
Total	*N*	270	60	234	141	224	123	1,052
	%	25.7%	5.7%	22.2%	13.4%	21.3%	11.7%	100.0%

1 *χ*^2^ = 145.2, df = 15, *p* = <0.001. % in bold type indicates cell significantly high at *p*  = 0.01 (if in parentheses, at *p*  = 0.05); % in underlined italics indicates cell significantly low at *p*  = 0.01 (if in parentheses, at *p*  = 0.05).

## Discussion

Using a large multinational sample, the findings illuminate both therapist trainees’ variable patterns of life quality and factors meaningfully associated with them. While not involving data on trainees’ educational attainments or treatment outcomes of their clients, important clinical questions and possible implications are raised by the findings.

### Life Quality Patterns

First, happily, it should be observed that more trainees than not experienced their lives as *Fortunate/Happy*, characterized by great or very great life satisfaction and less than great life stress. While little previous research exists on therapist trainees’ life satisfaction, the findings fit broadly with a recent study that included measures of life satisfaction and distress, investigating relatively experienced therapists (practice experience level *M* = 13 years), and finding 52% characterizable as well-adapted or high functioning (Laverdière et al., 2018). The most worrisome group in the present study, the 14.3% of trainees in the *Distressed/Troubled* group, also broadly fit with the 10% of experienced therapists in the cited study who were characterized by significant psychological distress. The educationally and clinically relevant concern is obviously how such distress may affect trainees’ learning outcomes (Bücker et al., 2018) or therapy practice, in case they currently provide therapy (Beaumont et al., 2016).

Notably, a much higher prevalence has recently been reported for professional burnout, in both experienced therapists (Westwood et al., 2017) and trainees (Kaeding et al., 2017): in fact, in approximately every other professional. Given the divergence in constructs and measures, it is difficult to evaluate whether some degree of burnout—originally operationalized as involving experiences of emotional exhaustion, depersonalization, and low personal accomplishment (Maslach and Jackson, 1981)—may have been experienced by the 14.3% of Distressed/Troubled trainees (i.e., currently experiencing little satisfaction and much stress), who seem the likeliest to suffer work-related distress. Similar questions might be asked about the 8.7% *Intense/Impassioned* trainees (i.e., experiencing both notable life satisfaction and life stress) or the 21.7% *Low-key/Subdued* trainees (i.e., experiencing both little satisfaction and little stress). How may the greater stress experienced by the *Intense/Impassioned* trainees may affect their capacity for learning or focusing on their clients’ matters; and likewise, how might the little satisfaction experienced by *Low-key/Subdued* trainees affect their capacity to convey optimism, confidence, and hope to their clients (Orlinsky, 2022)? Indeed, this last question gives reason for looking more closely at what were shown to be the correlates of trainees’ life satisfaction and stress.

### Correlates of Life Quality Patterns

First, gender differences were essentially non-existent for life satisfaction, in line with prior literature (Diener et al., 1999; Geerling and Diener, 2020). Previous research has also found life satisfaction to often follow a U-shaped curve with age, with happiness levels reaching a low sometime in the 40s (Diener et al., 2018). Our results supported that partially, in that older therapists were most often *Fortunate/Happy*; and perhaps mirroring also cross-cultural studies on personality development, indicating that people tend to become lower in neuroticism (or negative affectivity) as they age (McCrae et al., 1999, 2000).

In line with early meta-analyses (Haring-Hidore et al., 1985; Wood et al., 1989), the findings also showed married trainees to be happier, although some research suggests that as people adapt to marriage, long-term life satisfaction may be little different than before it (Luhmann et al., 2012). At any rate, however, the freedom of single or unattached trainees did not seem to add to their life quality but rather vice versa, in line with prior studies (Myers, 2000). Another finding in line with prior literature (Diener et al., 2018) was that trainees’ current economic circumstances were an unequivocal correlate of life satisfaction. Of the trainees experiencing moderately to very difficult finances, one of two were either *Distressed/Troubled* or *Low-key/Subdued*. These findings are important from a policy perspective, as they underline how societal factors such as availability of affordable student loans and employment situation contribute to life satisfaction and stress (Diener et al., 2018).

Aspects of personality, as expressed in close personal relationships, also correlated significantly, and in expectable ways, with trainee life quality. The strongest correlate was for Genial/Caring style (*ρ* = 0.26), which corresponded in size with the second-strongest Big 5 personality trait correlate of life satisfaction from meta-analyses, *Extraversion* (*r* = 0.28; Steel et al., 2008). Indeed, a Genial/Caring or *self-bestowing* self-experience may overlap (1) with Extraversion in terms of a generally friendly and open disposition toward others, and (2) with another Big 5 personality correlate of life satisfaction, i.e., *Agreeableness* (*r* = 0.14), a warm, kind, and cooperative disposition (Steel et al., 2008). The capacity for trusting relationships emerged as significant but somewhat less important correlates both life satisfaction and stress, as Life Satisfaction was associated negatively (*ρ* = −0.16) and Life Stress positively (*ρ* = 0.19) with being Reclusive/Remote or *self-protective* in close personal relationships. Greater Anxious attachment and Avoidant attachment were also linked to greater Life Stress (*ρ* = 0.25 and 0.13) and lower Life Satisfaction (*ρ* = −0.26 and − 0.18). Recalling that Secure attachment is the opposite of anxious and avoidant attachment, these correlations show how much a capacity for more Secure attachment (e.g., as reflected in marital status) may help individuals attain a positive quality of life.

While the findings above are in line with studies indicating the contribution of (perceived) social relationships and support for life satisfaction (Geerling and Diener, 2020), it should be noted that the mechanisms linking personality and life satisfaction are still not well understood (Diener et al., 2018). For instance, given that self-report measures are often used for assessing both constructs, as also in the present study, it is not clear whether more open, sociable, or trusting people are better able to form relationships and that increases their life satisfaction; or, whether certain people have an overall tendency to see their life, personality, and relationships in more positive terms (Steel et al., 2008; Diener et al., 2018). The observed associations in the present and prior studies nevertheless suggest that creating a trusting and positive group atmosphere in therapist training might benefit both trainees’ life satisfaction but also their learning outcomes (Clément et al., 1994; Bücker et al., 2018; Reschke et al., 2021).

It is important to recognize how current adult life quality may also be influenced by earlier life circumstances. In the present study, trainees’ poorer life quality was notably associated with poorer family economic background as well as poorer family psychological and emotional functioning. This fits well with large-scale longitudinal studies showing adverse childhood experiences—including both financial strain as well as psychological and emotional dysfunction—predict severely reduced life quality (Nurius et al., 2015; Mosley-Johnson et al., 2019). From an intervention perspective, the important question then is which resilience resources may counteract these risks—such as having a sense of community, social integration, and emotional and social support, which have been found to beneficially moderate the effects of early adverse experiences in adulthood (Nurius et al., 2015). Indeed, an obvious further resource which may offer these social and emotional benefits are supervisory support and personal therapy, an often though not always required feature of training programs. Given the remarkable prevalence of early adversities and trauma in therapists (Pope and Feldman-Summers, 1992; Feldman-Summers and Pope, 1994; Orlinsky, 2022), this part of training may well be valuable and is also frequently rated by therapists as being so (Orlinsky and Rønnestad, 2005).

Lastly, in terms of social, cultural, and national origins of life quality, we also found that trainees who recognize they would be viewed in their society as a minority person are less often *Fortunate/Happy* and more often experiencing great stress (although also life satisfaction), tending to have a more *Intense/Impassioned* life. This finding was also in line with prior literature, which has found both ethnic and sexual minorities to experience greater stress (Cochran et al., 2003; Ramirez and Paz Galupo, 2019). Apart from its general policy making implications on a societal level, therapist training programs would also do well to attend to their trainees’ minority experience, and thus promote inclusiveness in both training and conduct of therapy (Davis et al., 2018).

In the same domain, we saw life quality to be notably associated with nationality itself. The most striking observation was the exceptionally frequent distress of Italian trainees. Although the direction of the findings fits with prior findings in comparison to some of other Western European countries, such as Austria, Germany, and Finland (Abdallah et al., 2008; Park et al., 2009), the size of the effect was nevertheless surprisingly large. As within-nation changes in happiness are typically also relatively small (Veenhoven and Hagerty, 2006), this finding may be partly attributable to confounding factors, e.g., greater financial distress among Italian trainees, which need further clarification.

### Methodological Limitations

The study involved a few major strengths. First, a comprehensive self-report instrument enabled covering a multitude of plausible determinants of life quality. Second, the large, multinational sample allowed identifying relatively subtle associations which, even if small, shed light on the nuances of trainees’ quality of life. Third, assessing both life satisfaction and stress allowed for a more balanced picture of life quality than assessing either one alone.

The study also involved some limitations common to life satisfaction research. First, the generalizability of the findings is unknown, since the sample was mostly based on convenience samples, representing ongoing training programs during the study years only in a few mostly European countries (e.g., Austria, Finland, and Italy), and even in these countries, percentages and data of non-responders are not available. However, the associations between life satisfactions and its correlates reflected largely findings of prior literature, supporting the validity and reliability of the present findings. Second, both life satisfaction and life stress were assessed as unidimensional measures, which might result in different answers in different contexts. However, studies have shown that even single-item measures in this area have moderately high reliability and validity, in addition to being most feasible for use in large-scale studies (Diener et al., 2018). Third, the cross-sectional design cannot establish temporal precedence on whether life satisfaction is preceded by some factors (e.g., marriage status), or vice versa, or if a third underlying variable is responsible for both of them. On the other hand, many of the variables in the present study reflect “objective” trainee characteristics (e.g., nationality, minority status, age) that would not be influenced by subjective life quality or reflect past situations (e.g., childhood economic background) that would not be influenced by current life quality, even if the mechanisms that link them to life satisfaction call for closer study. Fourth, relatedly, measurement issues have been raised regarding the common practice of assessing life satisfaction and its determinants retrospectively and by the same person (Nivison et al., 2021). While daily experience-sampling and day-reconstruction procedures have been suggested for achieving a more refined picture of experienced life satisfaction, these solutions remain labor-intensive and not feasible to implement in all research contexts (Diener et al., 2018).

### General Implications and Future Perspectives

There is sometimes a popular if implicit assumption that as the professionals of mental health, therapists must have the secret to the “good life” and thus also possess it. Equally, there may sometimes be a moralistic assumption that therapists *should* have it—for if one is not capable of living well oneself, how can one help others to do so? Yet the present study on therapist trainees indicates that even if they are a highly self-selected group of people by virtue of their preferred profession—and this self-selection may also be evidenced in some of their personality-related or relational qualities (Peter et al., 2017; Peter and Wolf, 2021)—therapist trainees appear subject to the same sources of life satisfaction and stress as people in general, just as good physicians can themselves be well or fall ill.

Given the findings that life satisfaction can substantially affect learning outcomes (Bücker et al., 2018), and more specifically in the case of therapists can affect their therapeutic relationships (Nissen-Lie et al., 2013), our results call for serious thinking about how to protect trainees’ life quality when it is good and how to raise it when it is deficient. As suggested in this study, some interventions may be targeted on a societal level (e.g., availability of low-cost student loans); some on a training program level (e.g., promoting supportive supervision and positive between-trainee relationships); and some on an individual level (e.g., providing personal therapy and learning positive self-care). Clearly further research is warranted to investigate how such interventions or even training curricula may promote a favorable balance trainees’ levels of life satisfaction and stress, and how both directly influence their learning and treatment outcomes.

## Data Availability Statement

The original contributions presented in the study are included in the article/supplementary files, further inquiries can be directed to the corresponding author.

## Ethics Statement

The studies involving human participants were reviewed and approved by Ethics Committee of the University of Witten/Herdecke. The patients/participants provided their written informed consent to participate in this study.

## Author Contributions

EH had the main responsibility for interpreting the results and writing the first and successive drafts of the manuscript. DO conceptualized the study, conducted the statistical analyses, contributed to the interpretation of the results, and participated in all stages of the study. UW was responsible for data management, AH for statistical consultation, and both participated in interpreting the results together with MR. EH, DO, UW, MR, TS, IM, HL-S, and AH all provided important intellectual content to the manuscript and study design. All authors contributed to the article and approved the submitted version.

## Conflict of Interest

The authors declare that the research was conducted in the absence of any commercial or financial relationships that could be construed as a potential conflict of interest.

## Publisher’s Note

All claims expressed in this article are solely those of the authors and do not necessarily represent those of their affiliated organizations, or those of the publisher, the editors and the reviewers. Any product that may be evaluated in this article, or claim that may be made by its manufacturer, is not guaranteed or endorsed by the publisher.
